# Sociodemographic and Health-Related Risk Factors Associated with Tooth Loss Among Adults in Rhode Island

**DOI:** 10.5888/pcd10.110285

**Published:** 2013-03-28

**Authors:** Yongwen Jiang, Catherine A. Okoro, Junhie Oh, Deborah L. Fuller

**Affiliations:** Author Affiliations: Catherine A. Okoro, Centers for Disease Control and Prevention, Atlanta, Georgia; Junhie Oh, Brown University School of Medicine and Rhode Island Department of Health, Providence, Rhode Island; Deborah L. Fuller, Rhode Island Department of Health, Providence, Rhode Island.

## Abstract

**Introduction:**

Oral health is an integral component of overall health and well-being. Very little Rhode Island state-level information exists on the determinants of tooth loss. The objective of this study was to systematically identify sociodemographic characteristics, health behaviors, health conditions and disabilities, and dental insurance coverage associated with tooth loss among noninstitutionalized adults in Rhode Island.

**Methods:**

We analyzed Rhode Island’s 2008 and 2010 Behavioral Risk Factor Surveillance System survey data in 2011. The survey had 4 response categories for tooth loss: none, 1 to 5, 6 or more but not all, and all. We used multinomial logistic regression models to assess the relationship between 4 risk factor domains and tooth loss.

**Results:**

An estimated 57.6% of Rhode Island adults had all their teeth, 28.9% had 1 to 5 missing teeth, 8.9% had 6 to 31 missing teeth, and 4.6% were edentulous. Respondents who had low income, low education, unhealthy behaviors (ie, were former or current smokers and did not engage in physical activity), chronic conditions (ie, diabetes and obesity) or disabilities, and no dental insurance coverage were more likely to have fewer teeth compared with their referent groups. However, the association of these variables with tooth loss was not uniform by age group.

**Conclusion:**

Adults who report risky health behaviors or impaired health may be considered target subpopulations for prevention of tooth loss and promotion of good oral health.

## MEDSCAPE CME

Medscape, LLC is pleased to provide online continuing medical education (CME) for this journal article, allowing clinicians the opportunity to earn CME credit.

This activity has been planned and implemented in accordance with the Essential Areas and policies of the Accreditation Council for Continuing Medical Education through the joint sponsorship of Medscape, LLC and Preventing Chronic Disease. Medscape, LLC is accredited by the ACCME to provide continuing medical education for physicians.

Medscape, LLC designates this Journal-based CME activity for a maximum of 1 **AMA PRA Category 1 Credit(s)™**. Physicians should claim only the credit commensurate with the extent of their participation in the activity.

All other clinicians completing this activity will be issued a certificate of participation. To participate in this journal CME activity: (1) review the learning objectives and author disclosures; (2) study the education content; (3) take the post-test with a 70% minimum passing score and complete the evaluation at www.medscape.org/journal/pcd (4) view/print certificate.

**Release date: March 27, 2013; Expiration date: March 27, 2014**

### Learning Objectives

Upon completion of this activity, participants will be able to:

- Analyze sociodemographic factors associated with tooth loss among adults
- Distinguish the most powerful sociodemographic predictor of tooth loss
- Evaluate the effect of physical activity on tooth loss
- Evaluate the effect of dental insurance coverage on tooth loss

**EDITORS**

Rosemarie Perrin, Editor, Ellen Taratus, Editor; *Preventing Chronic Disease*. Disclosure: Rosemarie Perrin and Ellen Taratus have disclosed no relevant financial relationships.

**CME AUTHOR**

Charles Vega, MD, Associate Professor and Residency Director, Department of Family Medicine, University of California-Irvine, Irvine. Disclosure: Charles P. Vega, MD, FAAFP, has disclosed no relevant financial relationships.

**AUTHORS AND CREDENTIALS**

Disclosures: Yongwen Jiang, PhD; Catherine A. Okoro, PhD, MS; Junhie Oh, BDS, MPH, have disclosed no relevant financial relationships. Deborah L. Fuller, DMD, MS served as an advisor or consultant for Metlife Priority Management Group.

Affiliations: Yongwen Jiang, Center for Health Data and Analysis, Rhode Island Department of Health and Brown University School of Medicine, Providence, Rhode Island; Catherine A. Okoro, Centers for Disease Control and Prevention, Atlanta, Georgia; Junhie Oh, Brown University School of Medicine and Rhode Island Department of Health, Providence, Rhode Island; Deborah L. Fuller, Rhode Island Department of Health, Providence, Rhode Island.

## Introduction

Oral health is an integral component of overall health and well-being (1). Poor oral health can lead to decreased general health, limited social functioning, and decreased quality of life (2,3). Tooth loss is an indicator of poor oral health and may impair physical, psychological, and social functioning and influence self-esteem and communication. People with tooth loss may avoid conversations or avoid laughing or smiling (2,4). Tooth loss also can affect nutrition, including impaired chewing ability, resulting in decreased intake of meat and firm fruit (2,5). Most tooth loss is due to dental caries and periodontal disease, although other causes include orthodontic or prosthetic treatment needs and traumatic injuries (3,6–14). Healthy People 2020 acknowledges the importance of maintaining permanent teeth by including an objective, OH-4, to decrease the proportion of adults who have ever had a permanent tooth extracted because of dental caries or periodontal disease (1).

Tooth loss is associated with smoking, inadequate oral hygiene, diabetes, hypertension, rheumatoid arthritis, depression, anxiety, obesity, anterior tooth type, and other risk factors including nutrition, alcohol consumption, socioeconomic status, lack of water fluoridation, and stress (3,6–14). Despite numerous studies and reports to determine risk factors related to oral health, research has not systematically explored the relationship between tooth loss and sociodemographic determinants, health behaviors, health conditions and disabilities, and access to dental care. The Behavioral Risk Factor Surveillance System (BRFSS) collects population-based information on many health domains, including sociodemographics, health behaviors, health conditions and disabilities, and health care access, and enables the evaluation of several risk factors simultaneously. However, very little Rhode Island state-level information exists on the determinants of tooth loss. The objective of this study was to systematically identify sociodemographic characteristics, health behaviors, health conditions and disabilities, and dental insurance coverage associated with tooth loss among noninstitutionalized adults in Rhode Island (RI).

## Methods

The BRFSS is an ongoing state-based surveillance system that uses standardized telephone surveys to assess the prevalence of key behavioral risk factors and chronic conditions among adults aged 18 years or older in all 50 states, the District of Columbia, and 3 US territories. Trained interviewers collect data monthly from an independent household probability sample drawn from the noninstitutionalized US adult population. In 2011 we analyzed data from the 2008 and 2010 Rhode Island BRFSS, which had a total sample size of 11,385 (4,786 in 2008 and 6,599 in 2010). Response rates, based on Council of American Survey Research Organizations guidelines, were 44.3% in 2008 and 47.6% in 2010, and a detailed description of the survey methods and questionnaire is available at www.cdc.gov/brfss.

### Measurement of tooth loss

Rhode Island BRFSS respondents were asked, “How many of your permanent teeth have been removed because of tooth decay or gum disease? Include teeth lost to infection, but do not include teeth lost for other reasons, such as injury or orthodontics.” Survey respondents were asked to choose from 1 of 4 tooth-loss response categories: none, 1 to 5, 6 or more but not all, and all (edentulous).

### Predictors

We chose 8 predictors of tooth loss on the basis of previous literature (2-3,5–14) and classified them into 4 domains (Box): sociodemographic status, health risk behaviors, health conditions and disabilities, and dental insurance coverage. Detailed definitions of the 8 predictors are available from www.health.ri.gov/data/details/definitions/behaviorrisksurveillancesystem.pdf.

**Box. Questions to Assess the 8 Predictors of Tooth Loss by 4 Domains**

**Sociodemographic Determinants**

Income: <$25,000; ≥$25,000

Education: High school degree or less; more than high school degree

**Health Risk Behaviors**

Cigarette smoking: Current smoker (smoked at least 100 cigarettes in lifetime and now smoke every day or some days); former smoker (smoked at least 100 cigarettes in lifetime but no longer smoke); never smoker (never smoked or smoked fewer than 100 cigarettes in lifetime)

Physical activity: Yes (participated in physical activity or exercise other than regular job such as running, calisthenics, golf, gardening, or walking during the past 30 days); no

**Health Conditions and Disabilities**

Diabetes: Has been told by a doctor that he/she has diabetes (gestational diabetes excluded); has not been told by a doctor that he/she has diabetes

Obesity: Not obese; obese, self-reported body mass index (weight in kilograms divided by square of height in meters) at or greater than 30 kg/m^2^

Disability: Yes (limited in any way in any activity because of any physical problem or using special equipment such as a cane, wheelchair, special bed, or special telephone); no

**Dental insurance coverage**

Dental insurance coverage: Yes (has any kind of insurance coverage that pays for some or all routine dental care, including dental insurance coverage, prepaid plans such as health maintenance organizations, or government plans such as Medicaid); no

### Statistical analysis

In our preliminary analyses, we included age, sex, income, education, employment status, race/ethnicity, marital status, and urban/rural residence, but only age, income, and education were significantly related to tooth loss. We excluded heart disease and stroke from the preliminary analyses because very few respondents reported either condition. In the preliminary analyses, we examined dental visits and dental insurance coverage as predictors of tooth loss as well, but only dental insurance coverage was significantly related to tooth loss. The final analyses were restricted to age, tooth loss, and the 8 predictors. Our preliminary analysis found that age was the strongest predictor for tooth loss; therefore, we stratified our analyses by 3 age groups (18–44 years, 45–64 years, and ≥65 years).

Multiple imputation has been used to simulate missing data in sample surveys. To retain all valid data and maintain maximal sample size, we handled missing data through multiple imputation according to the methods of Jiang and Hesser (15). We calculated prevalence estimates and χ^2^ statistics to identify significant associations between the 8 predictors and tooth loss. By using multinomial logistic regression, we also calculated adjusted odds ratios (AORs) and 95% confidence intervals (CIs) to assess the strength of the relationship between each predictor and the extent of tooth loss. The “0 missing teeth” group was the reference used to evaluate the potential risk effect of the 8 predictors. We also adjusted by age (treated as a continuous variable) within the age-stratified multivariate model, even though the analysis was age-stratified. We used 2-sided significance tests in all analyses. For all analyses, we considered only *P* values less than .05 significant. We used the PROC SURVEYFREQ and SURVEYLOGISTIC of SAS version 9.2 (SAS Institute Inc, Cary, North Carolina) to account for the complex survey design of the BRFSS.

## Results

An estimated 57.6% of Rhode Island adults had all their teeth, 28.9% had 1 to 5 missing teeth, 8.9% had 6 to 31 missing teeth, and 4.6% were edentulous. Increasing trends exist between demographic characteristics, risk factors, and extent of tooth loss across age groups except for 0 missing teeth among adults aged 65 years or older (Table 1).

**Table 1:** Prevalence of Tooth Loss by Demographic Characteristics and Risk Factors, by Age Group, Rhode Island BRFSS Respondents (N = 11,385), 2008 and 2010^a^^,^ b

Demographic Characteristic and Risk Factor	n (%)	%											
		18–44 y (n = 2,896), No. of Missing Teeth				45–64 y (n = 4,743), No. of Missing Teeth				≥65 y (n = 3,624), No. of Missing Teeth			
		0	1–5	6–31	All	0	1–5	6–31	All	0	1–5	6–31	All
**All respondents**	11,385 (100.0)	77.5	20.2	1.9	0.4	48.1	38.1	10.0	3.8	22.4	34.6	25.9	17.1
**Annual income, $**													
<25,000	2,619 (21.4)	62.9	32.5	4.0	0.7	26.5	39.2	23.2	11.0	17.1	25.6	29.2	28.1
≥25,000	7,146 (78.6)	80.8	17.6	1.4	0.2	51.9	37.9	7.8	2.4	25.0	39.4	24.2	11.3
**Education**													
≤High school degree	4,333 (36.1)	67.0	29.1	3.1	0.8	32.7	41.2	17.1	9.0	17.6	28.4	29.5	24.5
>High school degree	7,028 (64.0)	82.9	15.6	1.3	0.2	55.1	36.8	6.7	1.4	27.0	40.6	22.6	9.8
**Smoking status**													
Never smoker	5,690 (55.2)	82.7	16.3	0.8	0.3	58.4	34.7	5.6	1.4	28.4	39.9	20.6	11.1
Former smoker^b^	3,963 (28.3)	73.3	23.5	3.2	0.1	43.0	42.7	10.7	3.7	17.8	31.7	31.1	19.4
Current smoker^b^	1,689 (16.5)	63.6	30.6	4.7	1.2	29.3	38.6	21.4	10.6	18.7	24.1	23.3	34.0
**Physical activity^c^**													
Yes	8,156 (75.8)	80.5	17.6	1.6	0.3	51.9	37.1	8.5	2.5	23.9	37.6	25.1	13.5
No	3,221 (24.2)	65.3	30.6	3.3	0.8	36.2	41.3	14.9	7.7	19.6	29.2	27.6	23.6
**Diabetes**													
No	10,176 (92.4)	77.7	20.1	1.8	0.4	49.9	37.8	9.0	3.2	23.4	36.6	24.4	15.6
Yes	1,202 (7.6)	69.1	23.2	5.6	2.1	29.3	41.5	20.1	9.2	17.7	25.6	32.8	23.9
**Obese**													
No (BMI <30 kg/m^2^)	8,171 (76.0)	79.0	19.2	1.5	0.3	52.0	36.6	8.6	2.9	23.8	36.1	23.8	16.4
Yes (BMI ≥30 kg/m^2^)	2,750 (24.0)	72.8	23.9	3.0	0.3	37.7	42.2	13.7	6.4	17.2	29.8	33.8	19.3
**Disability**													
No	8,258 (79.1)	79.7	18.6	1.4	0.3	53.3	36.9	7.4	2.5	25.4	35.8	24.0	14.8
Yes	3,078 (20.9)	61.8	31.2	5.7	1.4	32.4	41.8	18.0	7.9	17.1	32.6	29.3	21.0
**Dental insurance coverage**													
Yes	6,837 (67.3)	80.4	17.6	1.6	0.4	51.4	38.4	8.1	2.2	26.0	40.0	25.2	8.8
No	3,995 (32.7)	70.8	26.2	2.7	0.3	38.1	38.3	15.3	8.3	19.9	31.6	26.4	22.2

Abbreviations: BRFSS, Behavioral Risk Factor Surveillance System; BMI, body mass index.

a All estimates were calculated using the raw data. All n values are unweighted; percentages are weighted. Subcategories may not sum to 11,385 because of missing values. Percentages may not sum to 100 because of rounding. We used χ^2^ test to test the difference in distribution of tooth loss by sociodemographics, health risk behaviors, health conditions and disabilities, and dental insurance coverage. All differences were significant (*P* < .001), except for diabetes (*P* = .02) and obesity (*P* = .01) among adults aged 18 to 44 years.

b Current smoker, defined as smoked at least 100 cigarettes in lifetime and now smoke every day or some days; former smoker, defined as smoked at least 100 cigarettes in lifetime but no longer smoke.

c Participated in physical activity or exercise other than regular job such as running, calisthenics, golf, gardening, or walking during the past 30 days.

Respondents who had low income, low education, unhealthy behaviors (ie, former or current smokers and did not engage in physical activity), chronic conditions (ie, diabetes and obesity) or disabilities, and no dental insurance coverage were more likely to have fewer teeth compared with their referent groups (Table 2). However, the association of these variables with tooth loss was not uniform by age group.

**Table 2:** Adjusted Odd Ratios of Tooth Loss for Demographic Characteristics and Risk Factors, Rhode Island Adults, 2008 and 2010^a^^,^ b

Demographic Characteristic and Risk Factor	n_1_/n_2_ c	AOR (95% CI)		
		1–5 Missing Teeth vs 0 Missing Teeth	6–31 Missing Teeth vs 0 Missing Teeth	Edentulous^d^ vs 0 Missing Teeth
**18–44 y**				
<$25,000 vs ≥$25,000	556/2,025	1.27 (1.07–1.50)	1.63 (1.10–2.40)	2.27 (1.20–4.30)
≤High school degree vs >high school degree	892/2,001	1.39 (1.20–1.61)	1.41 (1.04–1.92)	1.66 (0.91–3.03)
Former smoker^e^ vs never smoker	542/1,789	1.03 (0.84–1.25)	1.33 (0.86–2.03)	0.39 (0.13–1.15)
Current smoker^e^ vs never smoker	556/1,789	1.33 (1.07–1.65)	1.99 (1.36–2.93)	3.18 (1.10–9.17)
No leisure time activity^f^ vs leisure time activity	630/2,263	1.22 (1.06–1.41)	1.19 (0.85–1.67)	1.12 (0.58–2.14)
Diabetes vs no diabetes	93/2,801	0.98 (0.75–1.28)	1.35 (0.80–2.30)	1.97 (0.51–7.66)
Obese (BMI ≥30 kg/m^2^) vs not obese (BMI <30 kg/m^2^)	693/2,079	1.09 (0.93–1.26)	1.33 (0.99–1.79)	0.98 (0.44–2.19)
Disability vs no disability	421/2,466	1.24 (1.02–1.52)	1.62 (1.12–2.34)	1.66 (0.94–2.91)
No dental insurance vs dental insurance	660/2,065	1.18 (1.00–1.38)	1.18 (0.83–1.67)	0.79 (0.42–1.51)
**45–64 y**				
<$25,000 vs ≥$25,000	841/3,405	1.20 (1.06–1.36)	1.61 (1.35–1.91)	1.52 (1.19–1.94)
≤High school degree vs >high school degree	1,551/3,187	1.27 (1.15–1.39)	1.66 (1.44–1.90)	2.52 (2.04–3.13)
Former smoker^e^ vs never smoker	1,674/2,233	1.08 (0.95–1.22)	0.95 (0.80–1.13)	0.93 (0.71–1.23)
Current smoker^e^ vs never smoker	818/2,233	1.35 (1.14–1.59)	2.50 (2.01–3.10)	3.51 (2.57–4.80)
No leisure time activity^f^ vs leisure time activity	1,248/3,494	1.09 (0.99–1.20)	1.11 (0.96–1.28)	1.28 (1.04–1.57)
Diabetes vs no diabetes	483/4,257	1.19 (1.01–1.39)	1.50 (1.21–1.85)	1.53 (1.18–2.00)
Obese (BMI ≥30 kd/m^2^) vs not obese (BMI <30)	1,278/3,284	1.14 (1.04–1.26)	1.22 (1.06–1.40)	1.42 (1.14–1.79)
Disability vs no disability	1,319/3,402	1.20 (1.09–1.32)	1.48 (1.27–1.71)	1.55 (1.26–1.89)
No dental insurance vs dental insurance	1,217/3,341	1.02 (0.93–1.13)	1.14 (0.98–1.33)	1.54 (1.25–1.90)
**≥65 y**				
<$25,000 vs ≥$25,000	1,208/1,679	0.98 (0.86–1.12)	1.12 (0.97–1.28)	1.40 (1.20–1.63)
≤High school degree vs >high school degree	1,850/1,762	1.08 (0.96–1.21)	1.40 (1.23–1.59)	1.70 (1.46–1.98)
Former smoker^e^ vs never smoker	1,704/1,604	1.20 (1.00–1.44)	1.47 (1.22–1.78)	1.26 (1.02–1.54)
Current smoker^e^ vs never smoker	302/1,604	0.87 (0.65–1.17)	1.18 (0.88–1.58)	2.20 (1.60–3.02)
No leisure time activity^f^ vs leisure time activity	1,309/2,311	0.95 (0.84–1.07)	0.97 (0.85–1.10)	1.09 (0.95–1.26)
Diabetes vs no diabetes	620/3,002	0.95 (0.81–1.11)	1.19 (1.02–1.39)	1.27 (1.06–1.52)
Obese (BMI ≥30 kg/m^2^) vs not obese (BMI <30 kg/m^2^)	773/2,737	1.04 (0.90–1.20)	1.30 (1.12–1.51)	1.20 (1.01–1.43)
Disability vs no disability	1,307/2,299	1.17 (1.04–1.32)	1.24 (1.09–1.40)	1.28 (1.11–1.47)
No dental insurance vs dental insurance	2,081/1,364	1.04 (0.94–1.16)	1.12 (0.99–1.27)	1.58 (1.36–1.84)

Abbreviations: AOR, adjusted odds ratio; CI, confidence interval; BMI, body mass index.

a All estimates were calculated by using the data after multiple imputation.

b Analyses were adjusted for age (continuous) and all other variables in the table, even though the analysis was age-stratified.

c n_1_ denotes the unweighted sample n in the risk group, and n_2_ denotes the unweighted sample n in the low-risk group (referent).

d Because of the small sample size of edentulism among adults aged 18 to 44 years, the 95% CIs of the AORs are wide and indicate potentially unstable estimates.

e Current smoker, defined as smoked at least 100 cigarettes in lifetime and now smoke every day or some days; former smoker, defined as smoked at least 100 cigarettes in lifetime but no longer smoke.

f Participated in physical activity or exercise other than regular job such as running, calisthenics, golf, gardening, or walking during the past 30 days.

The likelihood of tooth loss increased when 6 of the 8 predictors of tooth loss (income and former smoker were not predictors) were present among adults aged 45 to 64 years (Table 2). Among adults aged 65 years or older, former smokers were more likely to report 6 to 31 missing teeth (AOR, 1.47; 95% CI, 1.22-1.78) and total tooth loss (AOR, 1.26; 95% CI, 1.02–1.54) than those who had never smoked. The strongest predictor of tooth loss among all age groups was current smoking status. In particular, among adults aged 45 to 64 years, current smokers were more likely to report tooth loss than those who had never smoked (1 to 5 missing teeth: AOR, 1.35; 95% CI, 1.14–1.59; 6 to 31 missing teeth: AOR, 2.50; 95% CI, 2.01–3.10; and total tooth loss: AOR, 3.51; 95% CI, 2.57–4.80). The same patterns also existed among the younger and older age groups. A significant relationship existed between no leisure time physical activity and 1 to 5 missing teeth in young adults but not among the other 2 age groups examined. Among middle-aged adults, those who had diabetes were more likely to report 1 to 5 missing teeth (AOR, 1.19; 95% CI, 1.01–1.39), 6 to 31 missing teeth (AOR, 1.50; 95% CI, 1.21–1.85) and total tooth loss (AOR, 1.53; 95% CI, 1.18–2.00) than those who did not have diabetes. Among middle-aged and older adults, those who had disabilities had an increasing trend in the odds of tooth loss compared with those who did not have disabilities. Not having dental insurance coverage was significantly related to complete tooth loss among middle-aged and older adults. Our data showed almost 1 in 3 Rhode Island adults lacks dental insurance coverage (32.7%) (Table 1), almost 4 times the percentage that lack medical insurance (8.6%) (calculated from 2008 and 2010 BRFSS data).

## Discussion

We found that the likelihood of having a risk factor increased with extent of tooth loss and that a dose–response relationship was maintained among middle-aged and older adults. The relationships between risk factors and tooth loss differed by age groups. For instance, we found a significant relationship between lower income and 1 to 5 missing teeth among young and middle-aged adults but not among older adults. Being a former smoker was significantly related to having lost 1 to 5 teeth in older adults but not among young and middle-aged adults.

Lower income and fewer years of education increase risk for oral disease (6,8,16,17). Our study confirmed these findings: people with low income (<$25,000 year) and low education levels (less than a high school degree) had a higher prevalence of tooth loss compared with their reference groups, although education was a stronger predictor than income. Americans living in poverty were 3 times as likely to have untreated dental disease than those who were not (18). People with higher incomes are more likely to have dental insurance coverage as a benefit and to practice oral disease prevention (19). People with low incomes have cost barriers to oral health care, are less likely to be aware of the need for comprehensive, ongoing dental care, and are more likely to use tobacco and have a poor diet (1). Our study found that education beyond high school was inversely associated with the number of teeth lost, even after controlling for other confounders. Those with higher education levels are usually employed, tend to have higher income, and have higher demand for and use of oral health services. Conversely, lower education results in lack of oral health knowledge, insufficient preventive behaviors, and low use of oral health services (8). Some have argued that access to dental care explains most of the socioeconomic disparities in oral health (6).

Smoking is an established risk factor for poor oral health (16). Cigarette smokers are more likely to have more missing teeth and to experience greater rates of tooth loss than nonsmokers (20,21). Our results are consistent with previous studies that link smoking status to tooth loss (22,23). In 2004, the US Surgeon General reported that sufficient evidence exists to infer a causal relationship between smoking and periodontal disease (21). Several hypothesized mechanisms underlie the relationship between smoking and oral health, including impairment of the immune system, alteration of the bacterial environment, increase of endodontic diseases, and decrease of salivary function (20). Millar and Locker found that current smokers were more likely to report oral health problems and less likely to use dental services than nonsmokers (2), which may decrease early-stage diagnosis of oral health problems (16). Cigarette smoking is a major modifiable risk factor for tooth loss. Smoking cessation can result in substantial improvements in oral health and could be an effective strategy to prevent tooth loss (2,21). To prevent periodontal disease, and, ultimately, tooth loss, dentists and other health practitioners have an important role to play in tobacco control, including provision of brief smoking cessation advice and supportive materials during regular dental and health care visits (2,16,24).

Physical activity may reduce periodontal disease risk (17,25). In our study, physical inactivity increased the likelihood of tooth loss, but most AORs were not significant. Data from the National Health and Nutrition Examination Survey show that physically active adults have a lower risk of periodontitis, and adults with periodontitis had elevated levels of C-reactive protein and white blood cell counts in the gingival crevicular fluid (25). The underlying mechanism associating physical activity with tooth loss may be through enhancement of a person’s immunological response (25).

One major complication of diabetes is periodontal disease, which in severe cases can lead to tooth loss (26,27). People with diabetes have a significantly higher prevalence of tooth loss (5,26,27). A relationship between diabetes and tooth loss is of public health interest (17). Our findings confirm previous studies supporting an association between diabetes and oral health problems (17,27). The compromised immune response associated with diabetes may increase susceptibility to oral disease; conversely, good oral health may aid in glycemic control (27). It is important to educate people with diabetes of their increased risk for tooth loss through multidisciplinary efforts. Diabetes control may be more important to maintaining a good periodontal condition than how long a person has been treated for diabetes (5).

Obesity has emerged as a significant predictor of periodontal disease, and body mass index may influence total tooth loss via an association with periodontal disease (5,10,20,28). Our results support findings fromother studies (5,7).

People with disabilities are at greater risk for tooth loss, which may further compromise their health (3). Our study showed that adults with disabilities were more likely to have tooth loss than those without disabilities. Poor oral hygiene and increased risk for oral diseases have been associated with limited manual dexterity; dry mouth caused by medication side effects; diet modification, such as high calorie/high sugar meal supplements and processed foods; and access to oral health care, such as the number of dental offices that are accessible to people with mobility limitations (3). Early recognition and professional intervention, including oral health education, can ameliorate many of these problems (3).

Regular dental visits, which are important for preventing tooth loss (27), can detect and treat periodontal disease at an early stage to alter its natural progression (19). However, preventive dental visits are restricted by costs (6). Surveys have reported that people with dental insurance coverage are more likely to report a recent dental visit (4,19). Lack of preventive care may reflect differences in the availability of dental care, the ability to pay for dental services, or other barriers to receipt of dental services (eg, transportation, accessibility, competing time demands) (2). Having no dental insurance coverage creates a financial access barrier to use of dental services (ie, routine dental examination and cleanings) and results in an increased risk of tooth loss.

This study has several limitations. First, because the BRFSS excludes institutionalized persons and those without landline telephones, it may underestimate the prevalence of tooth loss among Rhode Island adults. Second, BRFSS is a cross-sectional study, so it cannot establish causal relationships. Third, low BRFSS response rates may relate to potential issues (eg, noncoverage bias), which are not unique to Rhode Island. However, previous studies have demonstrated that BRFSS estimates are reliable, valid, and are comparable to other population surveys (29). Additionally, although agreement exists between data on tooth loss based on self-reports and data based on clinical records, the potential for misclassification bias exists (30,31). Fourth, the BRFSS tooth-loss question assesses the number of teeth removed because of dental decay or gum disease but not for other reasons, such as injury or orthodontics. Thus, an inherent limitation exists in that survey respondents may not have differentiated actual cause of tooth loss in their response, resulting in an underestimation or overestimation of tooth loss. Finally, several previously reported predictors of tooth loss were not available for inclusion in this analysis, such as anterior tooth type, inadequate oral hygiene, hypertension, nutrition, stress level, and fluoridated water consumption (6,9,10,13,14). The BRFSS includes some questions every year and others in alternate years; oral health questions are asked in even years only. Therefore, we used the most recent oral health data available (ie, 2008 and 2010) to identify risk factors in 4 domains that were significantly associated with tooth loss.

Our study also has several strengths. First, although numerous studies (2–8,10,12,16,17,19,25–27,32) have examined the relationship between these predictors and oral health, they examined the association between only 1 to 3 risk factors and oral health (2,3,6,7,12,17,26,27,32). Our study focused on a broad range of health-related risk factors related to tooth loss. Many factors are highly correlated and cannot be understood independent of other factors. When we focused on 1 factor associated with tooth loss, we controlled all other risk factors. Second, our study used different outcomes than those used in previous studies (3–6,8,10,12,16,19,25,26,32). Furukawa et al (5) used “depth of periodontal pockets,” Kelbauskas et al (32) used “teeth surfaces with carious lesions,” Sanders et al (25) used “periodontitis case,” and Dye et al (16) used ”perceived dental treatment needs” as outcomes. Many of the studies tended to dichotomize oral health (2,3,6,7,12,26), categorizing adults as having or not having oral disease. We used 4 predefined response categories for tooth loss. This enabled us to examine the trend between health risk factors and the 4 categories of tooth loss among Rhode Island adults. Our study showed the same pattern for all the risk factors and tooth loss among adults aged 45 to 64 and 65 or older; the likelihood of self-reported risk factors increased with the level of respondent’s tooth loss. Third, our study is different from previous studies in population perspective and study design. Our study was a cross-sectional analysis representative of Rhode Island community-dwelling adults aged 18 years or older, whereas other studies were representative of the entire US adult population; were disease-, occupation-, or age cohort–specific; were conducted among non-US populations; or had another study design (4,5,10–12,20,24,27,32).

Our findings may be generalized to adult populations beyond this study and suggest that targeting interventions at high-risk groups is likely to improve oral health. Dentists and hygienists can provide education to patients to improve awareness of the tooth loss effects of smoking, lack of physical activity, and other negative health conditions (16). Health promotion counseling should include the prevention and control of oral disease risk factors and the maintenance of good oral health (17). The Rhode Island Oral Health Program can support and increase public awareness efforts to educate families about the importance of oral health as a part of overall health and well-being and can support increased access to preventive oral care through a dental home to reduce health disparities, especially for at-risk populations.

## Figures and Tables

**Table:** 

**1. The activity supported the learning objectives.**				
**Strongly Disagree**				**Strongly Agree**
1	2	3	4	5
**2. The material was organized clearly for learning to occur.**				
**Strongly Disagree**				**Strongly Agree**
1	2	3	4	5
**3. The content learned from this activity will impact my practice.**				
**Strongly Disagree**				**Strongly Agree**
1	2	3	4	5
**4. The activity was presented objectively and free of commercial bias.**				
**Strongly Disagree**				**Strongly Agree**
1	2	3	4	5

## Post-Test Information

To obtain credit, you should first read the journal article. After reading the article, you should be able to answer the following, related, multiple-choice questions. To complete the questions (with a minimum 70% passing score) and earn continuing medical education (CME) credit, please go to http://www.medscape.org/journal/pcd. Credit cannot be obtained for tests completed on paper, although you may use the worksheet below to keep a record of your answers. You must be a registered user on Medscape.org. If you are not registered on Medscape.org, please click on the "Register" link on the right hand side of the website to register. Only one answer is correct for each question. Once you successfully answer all post-test questions you will be able to view and/or print your certificate. For questions regarding the content of this activity, contact the accredited provider, CME@medscape.net. For technical assistance, contact CME@webmd.net. American Medical Association's Physician's Recognition Award (AMA PRA) credits are accepted in the US as evidence of participation in CME activities. For further information on this award, please refer to http://www.ama-assn.org/ama/pub/category/2922.html. The AMA has determined that physicians not licensed in the US who participate in this CME activity are eligible for **AMA PRA Category 1 Credits™**. Through agreements that the AMA has made with agencies in some countries, AMA PRA credit may be acceptable as evidence of participation in CME activities. If you are not licensed in the US, please complete the questions online, print the AMA PRA CME credit certificate and present it to your national medical association for review.

## Post-Test Questions

### Article Title: An Algorithm That Identifies Coronary and Heart Failure Events in the Electronic Health Record

**CME Questions**

1. You are seeing a 60-year-old woman with several medical problems. Her dentist has provided a preoperative form for you prior to a planned extraction of 2 teeth, and you are concerned with this patient’s oral health. In the current study by Jiang and colleagues, which of the following variables was most associated with having fewer teeth?
  - Lower educational attainment
  - Single, divorced, or widowed status
  - Rural vs urban residence
  - A history of alcohol abuse
2. Which of the following variables was strongest in predicting tooth loss in the current study?
  - Lower educational attainment
  - Lower income
  - Current smoking status
  - The absence of dental insurance
3. The patient in Question # 1 does not exercise. Based on the current study, what should you consider regarding the relationship between physical activity and the risk of tooth loss?
  - Physical activity was not associated in the risk of tooth loss in any study analysis
  - Reduced amounts of physical activity increased the risk of tooth loss among all patient groups
  - Physical activity predicted tooth loss only among older adults
  - Physical activity predicted tooth loss only among younger adults
4. The patient lacks dental insurance. In the current study, what role did dental insurance have in promoting tooth loss?
  - Dental insurance was not associated in the risk of tooth loss in any study analysis
  - Dental insurance was the most important variable associated with tooth loss
  - A lack of dental insurance coverage predicted tooth loss only among middle-aged and older adults
  - A lack of dental insurance coverage predicted tooth loss only among younger adults

**Evaluation**
